# Effect of Different Anesthesia and Puncture Methods of Percutaneous Kyphoplasty on More Than 90-Year-Old Osteoporotic Vertebral Fracture: Advantages of the ERAS Concept

**DOI:** 10.1155/2022/7770214

**Published:** 2022-05-09

**Authors:** Hao Liu, Lei Deng, Jun-xin Zhang, Quan Zhou, Zhong-lai Qian, Chun-yang Fan, Kang-wu Chen, Hui-lin Yang

**Affiliations:** Department of Orthopaedics, The First Affiliated Hospital of Soochow University, Suzhou 215006, China

## Abstract

**Background:**

Percutaneous kyphoplasty (PKP) is an effective minimally invasive technique for the treatment of osteoporotic vertebral fracture (OVF) in recent years. This study focuses on the analysis of PKP surgery and anesthesia in osteoporotic vertebral facture patients over 90 years old with the concept of “enhanced recovery after surgery.”

**Methods:**

This study reviewed 239 patients who were diagnosed with OVF retrospectively between October 2015 and June 2019. According to the method of anesthesia, these patients were divided into Group A (*n* = 125) and Group B (*n* = 114). According to the pedicle puncture approach, these patients were divided into Group C (*n* = 102) and Group D (*n* = 137). The anterior vertebral height (AVH) and local kyphosis angle (LKA) were used to evaluate the degree of vertebral damage and restoration. The visual analogue scale (VAS) and the Oswestry Disability Index (ODI) scores were used for assessing functional outcomes. Some parameters were used to assess the perioperative conditions such as operation time, amount of bone cement perfusion, intraoperative fluoroscopy times, anesthesia recovery time, time out of the bed, hospital stay, hospitalization cost, and complications.

**Results:**

The visual analogue scale (VAS), Oswestry Disability Index (ODI), anterior vertebral height (AVH), and local kyphosis angle (LKA) 1 day, 1 year after surgery, and at the last follow-up all showed significant improvement (*P* < 0.05) in comparison with those before surgery both in Groups A and B and Groups C and D. The ODI 1 day after surgery was significantly better in Group B than Group A (*P* < 0.05). Compared with Group B, Group A required longer time of anesthesia, operation time, anesthesia recovery time, time to get out of bed, and length of hospital stay and more hospitalization costs (*P* < 0.05). Group D required longer operation time, longer time to get out of bed, more bone cement volume, fluoroscopy time, and more operation hospitalization costs compared with Group C (*P* < 0.05).

**Conclusion:**

We recommend unilateral puncture under local anesthesia for OVF in the patients aged over 90 from the perspective of rapid recovery.

## 1. Introduction

In 2000, China's fifth census showed that the population aged 60 and above accounted for 10.33% of the total population. Thus, China has entered a population aging society. With the progress of the past decade, in 2019, the population aged 60 and above accounted for 17.9% of the total population in China and the population aged 65 and above accounted for 11.9%. With the aging of the population, bone mass loss and the prevalence of osteoporosis will continue to increase, suggesting that the elderly in China are facing a high risk of osteoporosis. Patients with osteoporosis are prone to osteoporotic fractures because of decreased bone mass, destruction of bone microstructure, increased bone fragility, and decreased bone strength [1–3]. One osteoporotic fracture occurs every 3 s worldwide, and approximately one-third of women and one-fifth of men above the age of 50 are at risk of suffering an osteoporotic fracture. According to the data of the Sixth National Census of China in 2010, the number of osteoporotic vertebral fracture (OVF) cases in China is 44.49 million and increasing by 1.81 million cases every year. It is equivalent to an additional osteoporotic vertebral fracture occurring every 17.4 s, resulting in a medical expenditure of up to 9.45 billion yuan [4].

Percutaneous kyphoplasty (PKP) was first designed by Wong and Reiley and was approved by the Food and Drug Administration for clinical use in 1998 [5]. Liberman first reported the use of PKP in the clinic in 2001 [6]. With the development of the minimally invasive spinal surgery, PKP has achieved satisfying pain relief and functional improvement in treating OVF [7], spinal metastatic tumors [8], hemangioma, myeloma, vertebral nonunion, and others [9]. PKP considerably improves patient life expectancy and greatly reduces the complications caused by the conservative treatment of OVF patients. PKP facilitates “one needle fracture,” minimally invasive reconstruction of the spinal sequence and re-erects the spine [10–13].

With the continuous improvement of medical conditions, the life expectancy of China's population continues to increase, and the number of patients above 90 years of age with OVF is increasing. We must pay attention to the surgical treatment of these patients. The special characteristics of patients above 90 years of age are basic diseases, poor cardiopulmonary function, poor surgical tolerance, and more preoperative fasting complications. In 1997, Professor Kehlet first proposed “fast-track surgery” [14]. In 2005, enhanced recovery after surgery (ERAS) was proposed by the European Committee on Clinical Nutrition and Metabolism to reduce perioperative complications and speed up postoperative recovery. ERAS refers to a series of optimization measures made in the perioperative period with supportive medical evidence to reduce the physical and psychological traumatic stress of the surgery patients, in order to achieve rapid recovery. The primary aim of this approach is to reduce surgical stress, oral analgesic drugs, and preoperative and postoperative supplementation of nutrient solutions and promote early postoperative activities to accelerate the early recovery of patients. Although originally developed for patients undergoing colorectal surgery, the principles of ERAS have been found applicable to a wider patient population over the past decade. In recent years, the ERAS concept has garnered considerable scientific attention in orthopedics [15–18]. Spine surgery is associated with high postoperative pain and morbidity, slow recovery of function, and long hospital stays. In spine surgery, the era's approach reduces surgical stress by improving anesthesia and surgical methods during the perioperative period and promotes early decompression function exercises for patients, thereby achieving early postoperative recovery. Evidence indicated that ERAS may lead to a reduction in the length of hospital stay, complications, and postoperative symptoms [19, 20]. We recognize that ERAS may be necessary for perioperative management of PKP in very old patients with the most clinical complications and longest postoperative recovery time. We hypothesized that superaged OVF patients with advanced age could achieve better recovery by simplifying anesthesia and reducing surgical time in PKP surgery.

To verify this hypothesis, we focused on analyzing the strategies and clinical efficacy and perioperative performance of different anesthesia and pedicle puncture methods of PKP for OVF patients with age more than 90 years.

## 2. Methods

### 2.1. Selection Criteria

The inclusion criteria are as follows: (1) all patients and their families signed informed consent forms, and approval was obtained from the ethics committee of hospital; (2) (OVF) was diagnosed by imaging examination results; (3) age ≥ 90 years; (4) the follow-up time was more than 2 years; (5) patients with single-level injured vertebral body.

The exclusion criteria are as follows: (1) patients with pathologic vertebral lesions such as vertebral metastatic carcinoma, vertebral hemangioma, and myeloma; (2) patients with spinal cord or nerve root compression; (3) patients who died or were unable to complete the final follow-up.

### 2.2. General Information

A total of 239 patients who were diagnosed with OVF between October 2015 and June 2019 were enrolled in this study. According to the method of anesthesia, these patients were divided into Group A and B (Group A: general anesthesia; Group B: local anesthesia). According to the pedicle puncture approach, these patients were divided into Group C and D (Group C: unilateral pedicle approach; Group D: bilateral pedicle approach).

### 2.3. Surgical Technique

The operation was performed under general or local anesthesia. The patient is placed in a prone position and routinely disinfected with a towel. First, locate the position of the injured vertebrae with a C-arm machine and mark in the patient's body surface. Make a 0.5 cm longitudinal incision along the mark and then puncture through a unilateral or bilateral pedicle. When the C-arm machine fluoroscopic needle tip reaches 3 mm of the anterior edge of the vertebral body, the core of the needle is pulled out. The working cannula is placed, followed by placing the balloon into the vertebral body through the working cannula, and the balloon is expanded under the guidance of fluoroscopy. Bone cement is then modulated, and the prepared bone cement is slowly pushed into the vertebral body through the cannula. After the C-arm machine sees that the bone cement penetrates well and there is no leakage in the fractured vertebral body, the cannula is pulled out and the operation is completed. After 12 h after surgery, the patient is allowed to get out of bed and walk.

### 2.4. Assessed Parameters

#### 2.4.1. Clinical Efficacy Parameters

Visual Analogue Scale (VAS) and the Oswestry Disability Index (ODI) were evaluated before surgery, 1 day and 1 year after surgery, and at the final follow-up. Patients' subjective pain perception was assessed by VAS score (0–10 scale, with 0 being painless and 10 being the most painful) [21]. In addition, ODI scores were used to evaluate improvements of patients' quality of life. The anterior vertebral height (AVH) and local kyphosis angle (LKA) of the injured vertebral body were measured by professional spinal surgeon before surgery, 1 day and 1 year after surgery, and at the final follow-up, to evaluate the degree of vertebral damage and restoration. The AVH was defined as the percentage of the anterior height of injured vertebral body with respect to the mean value of anterior vertebral height of the upper and lower injured vertebral body. The local kyphotic angle (LKA) was defined as the angle between the superior and inferior endplate of the injured vertebral body [22].

#### 2.4.2. Operational Indicators

Some parameters were used to assess the perioperative conditions, such as the operation time, amount of bone cement perfusion, intraoperative fluoroscopy times, anesthesia recovery time, time out of the bed, hospital stay, hospitalization cost (all the instruments and materials used in the operation were imported), and complications. Severe complications include respiratory depression, heart failure, hypertension and hypotension, lung disease, and delirium.

### 2.5. Statistical Methods

SPSS 26.0 software was used for data analysis in this study. Statistic values are expressed as mean ± standard deviation. The paired sample *t*-test and the independent sample *t*-test were used to compare various parameters in the same group. The *χ*^2^ test and the Fisher exact test were used for categorical variable data. *P* < 0.05 indicated statistical significance.

## 3. Results

### 3.1. Demographics

The demographic data of the two study groups are shown in Table 1. The mean age of the 239 patients was 92.40 ± 2.51 years (range, 90∼102), and the mean follow-up time was 24.92 ± 10.47 months (range, 12∼48). According to the method of anesthesia, these patients were divided into two groups: Group A (*n* = 125, mean age: 92.57 ± 2.55 years; mean follow-up time: 24.84 ± 10.54 months) and Group B (*n* = 114, mean age: 92.21 ± 2.45 years; mean follow-up time: 24.56 ± 11.05 months). No significant differences were observed between Groups A and B in terms of gender, age, bone mineral density (BMD), operation method, and basic diseases of the elderly such as hypertension, coronary heart disease, and chronic respiratory diseases (*P* > 0.05). According to the pedicle puncture approach, these patients were divided into two groups: Group C (*n* = 102, mean age: 92.55 ± 2.62 years; mean follow-up time: 25.50 ± 10.51 months) and Group D (*n* = 137, mean age: 92.29 ± 2.42 years; mean follow-up time: 24.12 ± 10.93 months). No significant differences were observed between Groups C and D in terms of gender, age, bone mineral density (BMD), operation method, and basic diseases of the elderly such as hypertension, coronary heart diseases, and chronic respiratory diseases (*P* > 0.05).

### 3.2. Clinical Efficacy Outcomes

**Figure 1:**
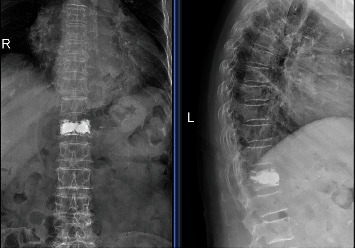
Anteroposterior and lateral radiographs of bilateral PKP at the follow-up after surgery.

**Figure 2:**
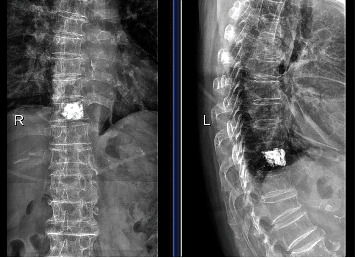
Anteroposterior and lateral radiographs of unilateral PKP at the follow-up after surgery.

The clinical efficacy data are shown in Table 2. The VAS, ODI, AVH, and LKA 1 day after surgery, 1 year after surgery, and at the last follow-up showed significant differences (*P* < 0.05) in comparison with those before surgery both in Groups A and B and Groups C and D. No significant differences were observed other than ODI in all preoperative and postoperative clinical efficacy indicators between Group A and Group B 1 day after surgery. Notably, the ODI of Group A (37.17 ± 2.81) was higher than that of Group B (35.95 ± 2.87) 1 day after surgery (*P* < 0.05). No significant differences (*P* > 0.05) were observed in all preoperative and postoperative clinical efficacy indicators between Group C and Group D (*P* > 0.05).  Figure 1 shows the radiograph of bilateral PKP after surgery.  Figure 2 shows the radiograph of unilateral PKP after surgery.

### 3.3. Operational Indicators and Complications

The operation-related indicators and complications are shown in Table 3. No significant differences were observed between Group A and Group B in operation time, cement volume, and fluoroscopy time (*P* > 0.05). Notably, significant differences were observed between Group A and Group B in anesthesia time, anesthesia recovery time, out of bed time, hospital stay, operation cost, and complications (*P* < 0.05). A total of 22 cases of complications were present in Group A, and the complication rate was 21.36%. A total of 3 cases of complications were present in Group B, and the complication rate was 2.70%. The complication rate in Group A was significantly higher than that in Group B (*P* < 0.05). Postoperative general anesthesia complications include postoperative vomiting, delirium, postoperative hypertension, heart failure, arrhythmia, refractory hyperglycemia, postoperative abdominal distention, and constipation. Postoperative local anesthesia complications include chest tightness, pain-induced convulsions, and nerve damage in puncture caused by convulsions. Notably, none of the patients in this study ended up choosing general anesthesia during surgery because they could not tolerate local anesthesia. At the same time, Group A had to pay more for hospitalization. No significant differences were observed between Group C and Group D in anesthesia time, anesthesia recovery time, hospital stay, and complications (*P* > 0.05). Notably, there were significant differences between Group C and Group D in operation time, cement volume, fluoroscopy time, time out of bed, and operation cost (*P* < 0.05).

## 4. Discussion

With the aggravation of the incidence of osteoporosis, the incidence of OVF in the elderly is considerably high. PKP for the treatment of OVF has good efficacy, especially for old patients, which can enable the patients to return to life in the early postoperative period and avoid related complications caused by bed rest. These complications even lead to the death of the patients. We found several studies on PKP in the treatment of OVF performed by domestic and foreign scholars [23, 24]; however, few analyses are available on the safety and effectiveness of PKP in the treatment of OVF in patients above 90 years of age. For superaged patients, research data are insufficient. We performed a systematic review of these patients to determine the correlation between perioperative treatment efficacy and surgical methods.

### 4.1. Summary of Research Results

No significant difference was found in demographic data between Groups A and B, as well as Groups C and D. VAS, ODI, AVH, and LKA after surgery significantly improved compared with those before surgery both in Groups A and B and Groups C and D. The ODI 1 day after surgery was better in Group A than in Group B. No significant differences were found between Groups A and B in clinical efficacy indicators. When we compared all preoperative and postoperative clinical efficacy indicators between Group C and Group D, no significant differences were observed. Compared with Group B, Group A required a longer time of anesthesia, operation time, anesthesia recovery time, time to get out of bed, length of hospital stay, and more hospitalization costs. Compared with Group C, Group D required longer operation time, longer time to get out of bed, more bone cement volume and fluoroscopy time, and hospitalization costs.

### 4.2. Selection of Anesthesia Methods

Many researchers [25, 26] have reported that PKP is an effective method for the treatment of senile OVF, which can expeditiously relieve pain and effectively recover the height of the vertebral body and Cobb angle. Liu et al. [27] reported that local anesthesia for PKP surgery in a single vertebral body was effective in relieving pain. Local anesthesia provides the same clinical benefits as general anesthesia. However, compared with PKP with general anesthesia, PKP surgery with local anesthesia can effectively reduce the cardiopulmonary system- and other anesthesia-related complications. Patients can get out of bed earlier, and hospital stay and surgical costs can be reduced. In this study, the VAS of PKP with local anesthesia and general anesthesia both significantly improved after surgery, and no significant difference was observed between the two types of anesthesia. This indicated that different anesthesia methods do not have a significant difference in their effect on postoperative pain relief. ODI of patients decreased significantly in both groups, indicating that PKP surgery timely solves the dysfunction of elderly patients with OVF. However, the improvement in ODI after surgery was better in patients with local anesthesia than those with general anesthesia. After 1 year and at the last follow-up, the difference disappeared, indicating that different anesthesia methods had no significant effect on the long-term functional recovery of old OVF patients. This may have a relationship with the fact that on the day before surgery, we asked our patients to do some simple limb exercises in the prone position on the bed for 1 h, three times a day. We believe that such functional exercises can adapt to the operative posture of PKP with local anesthesia in advance. Thus, the patients will be more adapted to the operation process, and active functional exercise will help them recover early after surgery. It can also explain why none of the patients in this study ended up choosing general anesthesia during surgery because they could not tolerate local anesthesia. The changes in AVH and LKA values before and after surgery indicated that PKP surgery can effectively promote the recovery of vertebral height and correction of kyphosis in old patients, which is of great importance to improving the postoperative quality of life of elderly patients with OVF. However, the type of anesthesia method selected for PKP surgery had little effect on this aspect. In general, PKP surgery with local anesthesia improved the clinical efficacy of OVF patients above 90 years of age more significantly. We believe that local anesthesia time is shorter, and patients do not need longer anesthesia waking time. Patients can come out of bed in a relatively short period of time, significantly reducing a series of complications caused by long-term bedding, at the same time reducing a series of risks of complications correlated with general anesthesia. This has obvious importance for the postoperative rehabilitation, early return to normal life, and improvement of life quality of old patients with OVF. Moreover, the hospitalization cost with local anesthesia is relatively less, which undoubtedly reduces the economic burden on the elderly. Many similarities are present between our results and theirs.

### 4.3. Selection of Puncture Methods

Many researchers [28–30] have reported that both bilateral and unilateral PKP are relatively safe and provide effective treatment for patients with painful OVF. However, unilateral PKP received less radiation dose and operation time, offered a higher degree of deformity correction, and resulted in fewer complications than bilateral PKP. By comparing the VAS, ODI, AVH, and LKA of unilateral and bilateral PKP before and after surgery, we found that pain, dysfunction, vertebral height recovery, and spinal deformity correction of elderly OVF patients were greatly improved after PKP surgery. Compared with bilateral PKP procedures, unilateral PKP procedures may achieve similar clinical efficacy. Because bilateral PKP requires repeated puncture assisted by fluoroscopy, it increases the operation time, radiation dose, and iatrogenic injury. For elderly patients with many complications, PKP surgery should be completed quickly and effectively. Compared with bilateral PKP surgery, unilateral PKP surgery is cost-effective because it uses fewer surgical instruments such as puncture needles and balloons. Thus, unilateral PKP surgery can reduce the financial burden on patients. At the same time, unilateral PKP surgery requires less cement, which can reduce cement leakage to a certain extent. Some researchers believe that the different distribution patterns of bone cement during unilateral and bilateral PKP surgery have different significance for postoperative pain relief. For instance, Liu et al. [31] reported that patients achieve more rapid pain relief with confluent rather than separated bilateral cement in PKP for OVF, with multiple linear regression analyses revealing that age and cement volume contributed to a rapid improvement of back pain. The authors believe that old people are more tolerant of pain after surgery than younger people. Therefore, we believe that unilateral PKP surgery has a less surgical effect on old patients with OVF, as it reduces iatrogenic injury, and that is more conducive to old patients returning to normal life as soon as possible. Thus, bilateral cement should be selected appropriately with due consideration to all relevant factors.

### 4.4. ERAS Concept Shows Advantages

ERAS is a multiprofessional and multidisciplinary approach to the care for surgical patients. This multimodal approach to recovery was first outlined by a Danish surgeon, Henrik Kehlet, in 1995 for colonic resection [32]. It is a medical model based on evidence-based medicine aimed to provide professional care and guidance for the whole perioperative period through the cooperation of medical staff of various disciplines. It can promote the recovery of patient physical function, reduce adverse psychology, improve compliance, reduce hospitalization costs, and speed up the recovery of patients [33]. ERAS represents reasonable, rapid, comprehensive, and reducing perioperative stress reaction and serious complications caused by long-term bed rest and provides patients with the best quality medical services [34]. In surgical practice, advanced age is an important risk factor for postoperative morbidity and mortality. To minimize postoperative complications and reduce the length of hospital stay, multidisciplinary approaches for the care of surgical patients, known as ERAS, are widely used [35, 36]. ERAS has a range of evidence-based care elements that support the body's response to stress caused by the injury. A reduction in these surgical stress responses is particularly important in vulnerable elderly patients with basic diseases [37]. Wainwright et al. [38] reported that spinal surgery is associated with considerable pain, slow recovery of function, prolonged hospital stay, and other complications. They suggest that spinal surgery under the ERAS principle may speed functional recovery and minimize postoperative complications. Unilateral PKP can reduce the anesthetic shock, minimize the operation time, and reduce the degree of trauma to achieve accelerated recovery of the elderly. For elderly patients with osteoporotic lumbar compression fractures, early regular core strength training positively affects early functional recovery and improvement of living ability after PKP, which is in line with the concept of ERAS [39]. There are differences in preoperative preparation between general and local anesthesia and unilateral and bilateral puncture. Preparation before general anesthesia and bilateral puncture should be more adequate. Fasting before general anesthesia poses a challenge to patients' tolerance and has a considerable effect on blood pressure and blood glucose levels in elderly patients. However, local anesthesia does not have absolute advantages. For example, the surgical experience of local anesthesia patients is poor and some patients complain of pain at the puncture site and chest tightness during surgery. Patients' restless movements may affect the surgical process by affecting the continuity of surgery. Therefore, we can also promote the early postoperative core muscle strengthening exercise in elderly patients to accelerate the postoperative rehabilitation of the elderly. Meanwhile, during the rehabilitation process after surgery, old patients should be actively treated with antiosteoporosis therapy. According to the present study, although the use of local anesthesia and unilateral puncture can reduce the cost of surgery and the financial burden on patients, at the social level, only prevention and medical strategies can address the increased cost and burden on healthcare for the elderly.

In our study, we only focused on the treatment of PKP under local anesthesia for single vertebral fractures better than general anesthesia. For multiple vertebral fractures, local anesthesia might not be suitable for all patients with OVF. Some researchers believe that general anesthesia has more advantages than local anesthesia [40]. In addition, to highlight the advantages and disadvantages of the two anesthesia methods, we only considered anesthesia-related complications and did not consider postoperative complications related to PKP surgery. At the same time, we did not make a detailed comparison of the specific costs of surgery (including surgical equipment, medication, nursing, examination, and other costs). During follow-up, there was no continuous monitoring of the patient's continued functional exercise after surgery.

## 5. Conclusions

PKP surgery in very old OVF patients warrants more attention with a focus on reducing the operation time as much as possible, expediting the postoperative recovery, and reducing surgical stress and shock resulting from simple anesthesia and puncture approach. This approach can help patients to return to their normal life at an early stage and will facilitate antiosteoporosis treatment and rehabilitation exercises after surgery. We believe that unilateral puncture under local anesthesia is as effective as bilateral puncture under general anesthesia. However, from the perspective of rapid recovery, we recommend unilateral puncture under local anesthesia for OVF in patients aged more than 90 years.

## Figures and Tables

**Table 1:** The demographic data of groups.

	General and local anesthesia			Unilateral and bilateral puncture		
	Group A	Group B	*P* value	Group C	Group D	*P* value
Number of patients	125	114	—	102	137	—
Gender (male/female)	40/85	41/73	0.518	32/70	49/88	0.478
Age (years)	92.57 ± 2.55	92.21 ± 2.45	0.261	92.55 ± 2.62	92.29 ± 2.42	0.434
BMD (T-score)	−3.13 ± 0.34	−3.08 ± 0.24	0.243	−3.18 ± 0.32	−3.09 ± 0.26	0.257
Follow-up (months)	24.84 ± 10.52	24.56 ± 11.05	0.842	25.50 ± 10.51	24.12 ± 10.93	0.327
OM, U/B	55/70	47/67	0.665	55/47	70/67	0.665
AM, G/L						
Comorbidity (*n*)						
Hypertension	36	30	0.668	28	39	0.863
CHD	17	12	0.335	13	21	0.592
Stroke	8	5	0.493	7	9	0.928
CRD	10	6	0.398	6	11	0.523
Diabetes	21	18	0.833	19	26	0.755

BMD, bone mineral density; CHD, coronary heart disease; CRD, chronic respiratory diseases; OM, U/B, operation method, unilateral/bilateral; AM, G/L, anesthesia method, general/local.

**Table 2:** The clinical efficacy outcomes of groups.

	General and local anesthesia			Unilateral and bilateral puncture		
	Group A	Group B	*P* value	Group C	Group D	*P* value
VAS						
Preop	8.23 ± 1.00	8.09 ± 1.05	0.295	8.27 ± 0.92	8.12 ± 0.99	0.247
Postop 1 d	2.08 ± 1.06^*∗*^	1.99 ± 0.97^*∗*^	0.503	2.01 ± 1.02^*∗*^	2.08 ± 1.02^*∗*^	0.596
Postop 1 y	2.06 ± 1.08^*∗*^	2.00 ± 0.99^*∗*^	0.636	2.01 ± 0.90^*∗*^	2.05 ± 1.04^*∗*^	0.749
Final	2.00 ± 1.02^*∗*^	1.93 ± 1.02^*∗*^	0.641	1.99 ± 0.91^*∗*^	2.04 ± 1.02^*∗*^	0.717

ODI						
Preop	84.40 ± 3.57	84.78 ± 3.23	0.389	84.32 ± 3.71	84.78 ± 3.18	0.311
Postop 1 d	37.17 ± 2.81^*∗*^	35.95 ± 2.87^*∗*^	0.001	37.17 ± 2.81^*∗*^	33.71 ± 3.30^*∗*^	0.478
Postop 1 y	32.90 ± 3.26^*∗*^	32.45 ± 3.25^*∗*^	0.295	32.20 ± 3.26^*∗*^	32.77 ± 3.32^*∗*^	0.186
Final	32.56 ± 3.32^*∗*^	32.77 ± 3.34^*∗*^	0.579	32.61 ± 3.32^*∗*^	32.93 ± 3.27^*∗*^	0.449

AVH (％)						
Preop	45.96 ± 5.10	45.21 ± 5.06	0.260	45.91 ± 5.31	45.44 ± 4.75	0.467
Postop 1 d	81.76 ± 6.31^*∗*^	80.88 ± 6.20^*∗*^	0.276	81.87 ± 6.63^*∗*^	80.95 ± 5.96^*∗*^	0.264
Postop 1 y	78.78 ± 6.05^*∗*^	77.95 ± 6.12^*∗*^	0.292	78.96 ± 6.12^*∗*^	77.96 ± 6.05^*∗*^	0.208
Final	77.15 ± 6.45^*∗*^	76.19 ± 6.17^*∗*^	0.237	77.37 ± 6.54^*∗*^	76.19 ± 6.13^*∗*^	0.154

LKA (°)						
Preop	27.68 ± 3.03	27.28 ± 3.02	0.310	27.19 ± 2.98	27.44 ± 3.14	0.524
Postop 1 d	13.12 ± 2.92^*∗*^	13.43 ± 2.90^*∗*^	0.419	12.35 ± 2.66^*∗*^	12.84 ± 2.64^*∗*^	0.155
Postop 1 y	15.06 ± 2.23^*∗*^	15.39 ± 2.09^*∗*^	0.237	15.14 ± 2.29^*∗*^	15.26 ± 2.08^*∗*^	0.675
Final	15.30 ± 2.23^*∗*^	15.12 ± 2.10^*∗*^	0.521	15.29 ± 2.26^*∗*^	15.16 ± 2.11^*∗*^	0.625

Preop, preoperation; Postop 1 d, one day after operation; Postop 1 y, one year after operation; Final, the last follow-up; VAS, visual analogue scale; ODI, Oswestry Disability Index; AVH, anterior vertebral height; LKA, local kyphosis angle. ^*∗*^ compared with preoperation, *P* < 0.05.

**Table 3:** The operational indicators and complications of groups.

	General and local anesthesia			Unilateral and bilateral puncture		
	Group A	Group B	*P* value	Group C	Group D	*P* value
AT (min)	34.02 ± 0.26	7.78 ± 0.14	<0.001	20.73 ± 3.42	21.05 ± 3.87	0.173
OT (min)	32.62 ± 5.26	31.78 ± 4.68	0.243	28.62 ± 5.47	47.49 ± 4.86	<0.001
CV (ml)	7.65 ± 1.21	7.48 ± 0.97	0.157	6.24 ± 0.85	9.46 ± 0.72	<0.001
FT	14.21 ± 1.53	14.86 ± 1.24	0.453	11.52 ± 1.48	19.53 ± 1.56	<0.001
ART (min)	51.88 ± 0.26	0	<0.001	52.46 ± 5.37	53.06 ± 6.21	0.436
TOB(h)	19.17 ± 0.33	3.32 ± 0.08	<0.001	17.61 ± 2.31	17.93 ± 2.84	<0.001
HS (day)	8.74 ± 0.11	4.77 ± 0.06	<0.001	5.58 ± 1.64	5.83 ± 2.03	0.253
HC (ten thousand yuan)	4.52 ± 0.39	4.07 ± 0.34	<0.001	3.74 ± 0.30	4.32 ± 0.38	<0.001
Complications	22	3	<0.001	9	16	0.476

AT, anesthesia time; OT, operation time; CV, cement volume; FT, fluoroscopy time; ART, anesthesia recovery time; TOB, time out of bed; HS, hospital stay; HC, hospitalization cost.

## Data Availability

Data are available upon request to the corresponding author.

## Abbreviations

OVF: Osteoporotic vertebral fracture
PKP: Percutaneous kyphoplasty
ERAS: Enhanced recovery after surgery
VAS: Visual analogue scale
ODI: Oswestry Disability Index
AVH: Anterior vertebral height
LKA: Local kyphotic angle.
